# Supplementary material for: From sequence to enzyme mechanism using multi-label machine learning
Source: BMC Bioinformatics. 2014 May 19;15:150. doi: 10.1186/1471-2105-15-150 (PMC4229970; doi:10.1186/1471-2105-15-150)
Supplement: Additional file 2 — Java code of ml2db. Additional file ml2db_code.tar.gz contains the Java source code to run the multi-label machine learning experiments and save the results to database. The code’s Javadoc is included. [file 1471-2105-15-150-S2.zip › additional file 2/ml2db/utils/doc/uk/ac/standrews/utils/main/webutils/XMLUtils.html]

XMLUtils


JavaScript is disabled on your browser.


- Overview
- Package
- Class
- Use
- Tree
- Deprecated
- Index
- Help

- Prev Class
- Next Class

- Frames
- No Frames

- All Classes

- Summary:
- Nested |
- Field |
- Constr |
- Method

- Detail:
- Field |
- Constr |
- Method


uk.ac.standrews.utils.main.webutils

## Class XMLUtils

- java.lang.Object
- - uk.ac.standrews.utils.main.webutils.XMLUtils

- ---

    

  ```
  public class XMLUtils
  extends java.lang.Object
  ```

  XML utilities. To parse simple XML etc.

  Version:
  :   12 Dec 2007

  Author:
  :   Luna De Ferrari luna.deferrari-at-ed.ac.uk

- - ### Constructor Summary

    Constructors

    Constructor and Description

    `XMLUtils()`
  - ### Method Summary

    Methods

    | Modifier and Type | Method and Description |
    | --- | --- |
    | `static java.lang.String` | `fromDOMtoFile(org.w3c.dom.Document DOMdocument, java.lang.String filePath)` From JTidy DOM to file |
    | `static java.lang.String` | `getXmlComment(java.lang.String text)` Returns a string formatted as xml comment: |
    | `static XmlNode` | `getXmlFromHTML(java.lang.String url, java.lang.String xhtmlFile)` |
    | `static XmlNode` | `getXmlFromReader(java.io.BufferedReader reader)` Get the XML as SimpleElement from a URL reader. |
    | `static org.w3c.dom.Document` | `tidyDOM(java.io.InputStream inputStream)` JTidy is designed to read-in typical-quality (that is to say, bad) HTML and output something cleaner (you have a choice of options), and also provides a DOM interface for traversing HTML documents that can be fed to an XML parser. |

    - ### Methods inherited from class java.lang.Object

      `equals, getClass, hashCode, notify, notifyAll, toString, wait, wait, wait`

- - ### Constructor Detail


    - #### XMLUtils

      ```
      public XMLUtils()
      ```
  - ### Method Detail


    - #### fromDOMtoFile

      ```
      public static java.lang.String fromDOMtoFile(org.w3c.dom.Document DOMdocument,
                                   java.lang.String filePath)
                                            throws java.lang.SecurityException,
                                                   java.io.FileNotFoundException
      ```

      From JTidy DOM to file

      Parameters:
      :   `DOMdocument` -
      :   `filePath` -

      Returns:
      :   the file path

      Throws:
      :   `java.lang.SecurityException`
      :   `java.io.FileNotFoundException`


    - #### getXmlComment

      ```
      public static java.lang.String getXmlComment(java.lang.String text)
      ```

      Returns a string formatted as xml comment:

      ```
       <!--  comment to be inserted -->
      ```

      Parameters:
      :   `text` - the comment to be inserted

      Returns:
      :   the comment surrounded by appropriate xml-comment markers.


    - #### getXmlFromHTML

      ```
      public static XmlNode getXmlFromHTML(java.lang.String url,
                           java.lang.String xhtmlFile)
                                    throws java.io.IOException
      ```

      Throws:
      :   `java.io.IOException`


    - #### getXmlFromReader

      ```
      public static XmlNode getXmlFromReader(java.io.BufferedReader reader)
                                      throws java.io.IOException
      ```

      Get the XML as SimpleElement from a URL reader. SimpleElement can then be
      used by a SimpleDOMParser.

      Parameters:
      :   `reader` -

      Returns:
      :   a tree of xml elements

      Throws:
      :   `java.io.IOException`


    - #### tidyDOM

      ```
      public static org.w3c.dom.Document tidyDOM(java.io.InputStream inputStream)
                                          throws java.lang.SecurityException,
                                                 java.io.FileNotFoundException
      ```

      JTidy is designed to read-in typical-quality (that is to say, bad) HTML
      and output something cleaner (you have a choice of options), and also
      provides a DOM interface for traversing HTML documents that can be fed to
      an XML parser.
      http://www.ibm.com/developerworks/xml/library/j-jtp03225.html

      Parameters:
      :   `inputStream` -

      Returns:
      :   a DOM representation of the HTML document in the input stream

      Throws:
      :   `java.io.FileNotFoundException`
      :   `java.lang.SecurityException`


- Overview
- Package
- Class
- Use
- Tree
- Deprecated
- Index
- Help

- Prev Class
- Next Class

- Frames
- No Frames

- All Classes

- Summary:
- Nested |
- Field |
- Constr |
- Method

- Detail:
- Field |
- Constr |
- Method
